# The Incubation Periods of Dengue Viruses

**DOI:** 10.1371/journal.pone.0050972

**Published:** 2012-11-30

**Authors:** Miranda Chan, Michael A. Johansson

**Affiliations:** 1 Department of Epidemiology, Rollins School of Public Health, Emory University, Atlanta, Georgia, United States of America; 2 Dengue Branch, Division of Vector-Borne Diseases, Centers for Disease Control and Prevention, San Juan, Puerto Rico, United States of America; University of Texas Medical Branch, United States of America

## Abstract

Dengue viruses are major contributors to illness and death globally. Here we analyze the extrinsic and intrinsic incubation periods (EIP and IIP), in the mosquito and human, respectively. We identified 146 EIP observations from 8 studies and 204 IIP observations from 35 studies. These data were fitted with censored Bayesian time-to-event models. The best-fitting temperature-dependent EIP model estimated that 95% of EIPs are between 5 and 33 days at 25°C, and 2 and 15 days at 30°C, with means of 15 and 6.5 days, respectively. The mean IIP estimate was 5.9 days, with 95% expected between days 3 and 10. Differences between serotypes were not identified for either incubation period. These incubation period models should be useful in clinical diagnosis, outbreak investigation, prevention and control efforts, and mathematical modeling of dengue virus transmission.

## Introduction

Dengue viruses (DENV) are a major cause of illness, hospitalization, and death throughout the tropical and subtropical regions of the world [1]. Despite the prevalence of DENV and the mosquito vectors, *Aedes aegypti* and *Aedes albopictus*, some components of the transmission cycle are not well defined. Here we focus on the extrinsic and intrinsic incubation periods of DENV infections. The extrinsic incubation period (EIP) is the viral incubation period between the time when a mosquito takes a viremic bloodmeal and the time when that mosquito becomes infectious. The intrinsic incubation period (IIP) is the time between a human being infected and the onset of symptoms due to the infection. These periods are important determinants of the temporal dynamics of DENV transmission and are therefore critical for clinical diagnosis, outbreak investigation, implementation of prevention and control programming, and mathematical modeling of DENV transmission.

The EIP begins with a mosquito taking an infectious blood meal from a viremic human host. DENV present in the blood meal then invades the midgut, replicates, and eventually disseminates throughout the mosquito, which becomes infectious once virus reaches the salivary glands, at which point the mosquito is infectious and has thus completed the EIP [2]. Since the early 1900s when the etiology of dengue was being investigated, the EIP has been recognized as an important component of DENV transmission dynamics [3]. Due to its known dependence on temperature [4], [5], the EIP plays an important role in efforts to understand the influence of weather and climate on the spatiotemporal dynamics of DENV transmission and to incorporate those effects into mathematical models of DENV transmission (e.g. [6], [7], [8], [9]).

The EIP is generally referenced as being 8–12 days [10], [11], based on two sets of experimental observations [12], [13]. In these experiments, no blood-fed mosquitoes were infectious until 8 days post exposure, but were infectious by 12 days post exposure. These observations have not however been incorporated into explicit statistical models, which have the advantage of being able to include cofactors, such as temperature, and to formally describe expected values, expected variability, and confidence in model parameters.

In humans, there are two periods of interest: the IIP, which marks the onset of symptoms as described above; and the latent period, the period between infection and the onset of infectiousness. The latter is another important determinant of transmission dynamics, but data is extremely sparse, so here we focus on the IIP as it is an important determinant of the temporal dynamics of human disease and may be used in a differential diagnosis, for example, for a traveler returning from a DENV-endemic area [11]. The IIP also provides a rough estimate of the latent period as most individuals have been noted to become infectious within a day before or after the onset of disease [12]. Like the EIP, the IIP varies and the ranges most cited in the literature are those of the World Health Organization [10], 4–10 days, and the Centers for Disease Control and Prevention [11], 3–14 days, but typically 4–7. Again, these range estimates are based directly on observations from a limited numbers of studies [12], [14], but statistical models such as those of Nishiura and Halstead [15] have the potential to provide a more complete description including estimates of uncertainty.

Here, we apply multiple Bayesian time-to-event models to the DENV incubation periods. Time-to-event models have the distinct advantage of being able to combine direct observations and censored observations. Direct observations of DENV incubation periods are unique to the IIP observations from the early 1900s when humans were experimentally infected and monitored for symptom onset [3], [12], [13], [14], [16], [17], [18]. Censored observations are more common and include maximum or minimum observations, rather than a precise time. For example, for a traveler becoming sick after a short stay in an endemic area, the actual IIP is unknown, but the maximum and minimum IIP are defined by his or her arrival and departure times, respectively, to the onset of illness.

These models also offer the opportunity to investigate other factors that may influence the incubation periods. Those factors may include viral characteristics such as the fitness of particular serotypes or genotypes [19], [20], [21] and the amount of virus to which the mosquito or human is exposed [5], [22]. Both vector and host characteristics may also play a vital role; mosquito competence can be highly variable even within a single species [23], [24] and human susceptibility may vary due to prior exposure and intrinsic genetic factors [25], [26]. Finally, as mentioned above, temperature influences the EIP; at higher temperatures within the viable temperature range of the vector, DENV replicates faster and the EIP is shorter [4], [5]. Here, we investigate the influence of temperature on the EIP and potential difference in EIP and IIP between the four DENV serotypes.

## Materials and Methods

### Data

Relevant literature was collected by searching the PubMed, Ovid, and the Armed Forces Pest Management Board Literature Retrieval System databases using combinations of search terms including *Aedes aegypti*, *Stegomyia fasciata* (previous name for *Ae. aegypti*), *Aedes albopictus*, dengue, experiment, import, incubation, transmission, temperature, and travel. We did not restrict the search based on time of publication or language. Further material was found by reviewing references from identified papers.

The moment when a mosquito becomes infectious is not directly observable, so observations of the EIP are restricted to the window between exposure(s) and transmission experiment(s), defined by a minimum and maximum EIP. For example, if a mosquito is shown to be infectious 10 days after exposure, the EIP must be between 0 and 10 days. If the same mosquito is tested at day 5 and does not transmit DENV at that time, the EIP is between 5 and 10 days. For each observation, the maximum EIP was defined as the time from the first infectious blood meal to the first successful transmission of DENV. If transmissibility was tested and never successful, the maximum EIP is unknown. The minimum EIP was the time from the last infectious blood meal to the last negative transmission experiment or zero if there were no negative transmission experiments.

Acceptable transmission assays involved the confirmation of transmission to a naïve individual as evidenced by the onset of dengue or by laboratory evidence of infection such as hemagglutination inhibition or plaque reduction neutralization assays. Because dengue is used as an indicator, there may be some false-negative tests resulting from asymptomatic infections. We initially assume that all negative tests are truly negative and revisit this assumption later.

Observations of the EIP were limited to those in which *Ae. aegypti* or *Ae. albopictus* were fed on viremic humans or non-human primates. We also excluded observations in which infection of the mosquito was attained by injection or by feeding on animal blood or artificial media seeded with DENV, as these may not realistically mimic natural transmission.

Temperature data were recorded for each EIP observation when available. For observations with no temperature data, we obtained temperature data for the location of the study at the time of year when the study was undertaken from the Climate Research Unit 30-year mean climatology dataset (CL 2.0) [27]. The available temperature data was used to calculate a spatially and seasonally matched mean temperature for each observation.

The IIP analysis was restricted to events in which humans became sick after being experimentally infected by *Ae. aegypti* or *Ae. albopictus* or after being naturally exposed to DENV within a defined period of time by travelling into or out of an area with ongoing DENV transmission. In this case, the end event, the onset of symptoms, was always observed, but the exact exposure time is only known in the case of experimental infections. In those cases, the IIP was directly observed and therefore uncensored. In other cases, the maximum and minimum IIP were defined as the time from the first and last potential exposures, respectively, to the onset of illness. For example, a traveler who became sick 3 days after returning from a 10-day trip may have been exposed at any time during the trip, so the IIP must be between 3 and 13 days.

Further ancillary data collected for the analyses included the serotype of virus when known. The data is available in Text S1.

### Statistical Analysis

The EIP and IIP data were both analyzed using censored time-to-event models. For the IIP observations with a single exposure and a known time of illness onset, the data are uncensored. For observations of EIP or IIP defined by an interval, the event is interval-censored, i.e. the event occurred sometime between the minimum and maximum times defined by the observations. Observations with only a minimum time are treated as right-censored data.

For each incubation period, we analyzed four common time-to-event models: exponential, Weibull, gamma, and log-normal. The specific formulations of each are given in Table 1. For each model, we assumed multiplicative hazards using linear covariates, defined by *βX*. For the EIP, we incorporated a covariate for temperature (*T*) to estimate the temperature sensitivity and a random effect (*z*) to control for inter-study (*i*) variation which may arise from unique study designs and unknown properties of a particular human, monkey, mosquito, or virus population:
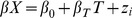
(1)


**Table 1:** Statistical distributions.

Distribution	Probability density function	Parameters	Covariates
Exponential	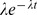	*λ* = rate	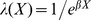
Weibull	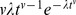	*λ* = rate	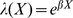
		*v* = shape	
Gamma	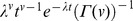	*λ* = rate	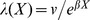
		*v* = shape	
Log-normal	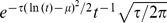	*μ* = mean	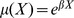
		*τ* = precision	

The IIP models only included the intercept *β*_0_ and random effect, not the temperature covariate. We also evaluated possible differences between serotypes using a dummy variable for each serotype.

We fitted the models using Markov Chain Monte Carlo methods. We used weakly informative priors for all coefficients (Text S2). To improve sampling, the random effects were hierarchically centered on the intercept estimate and the temperature variable was mean-centered. Thus equation 1 becomes:
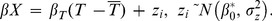
(2)


The *β_0_* coefficient that we report is adjusted for this and the between-study variation so that it may be used directly without centering: 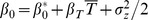. The IIP is only adjusted for between-study variation: 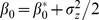. The between-study variation adjustment is necessary because *e^βX^* is log-normally distributed such that this variance contributes to the expected mean.

We initialized three Markov Chain Monte Carlo chains for each model and ran them until convergence based on visualization and the Gelman-Rubin statistic [28]. We then continued sampling, using thinning to reduce first-order autocorrelation to below 0.1, until we had at least 1,000 independent samples for estimation of the posterior distributions. Model fit was compared using the deviance information criterion (DIC) [29]. The DIC rankings were further assessed via bootstrapping (Text S3). The analyses were performed in R 2.15.0 (www.r-project.org). OpenBUGS Version 3.2.1 [30] using R2OpenBUGS [31] (www.openbugs.info).

## Results

### Data

We identified 38 studies reporting relevant observations of natural EIP and IIP [3], [5], [12], [13], [14], [16], [17], [18], [32], [33], [34], [35], [36], [37], [38], [39], [40], [41], [42], [43], [44], [45], [46], [47], [48], [49], [50], [51], [52], [53], [54], [55], [56], [57], [58], [59], [60], [61]. The crude data and further details regarding selection or exclusion of specific observations can be found in Text S1.

The EIP data included 146 observations from 8 studies published between 1905 and 1987 [3], [5], [12], [13], [14], [16], [32], [33]. In 109 instances, the EIP observation was interval-censored and in 37, right-censored. For 5 studies including 27 observations, serotype was unknown. The other three studies consisted of DENV-1 (49 observations), DENV-2 (38 observations), and DENV-4 (32 observations). *Ae. aegypti* were used for 140 of the observations and *Ae. albopictus*, for 6. The average temperature was reported for 54 observations and estimated for the remaining 92 based on average climate for the particular location and time of year. Overall, the average temperature ranged from 16.4 to 35.0°C with a median of 26.5°C.

For the IIP, 204 observations were collected from 35 studies published between 1903 and 2011 [3], [12], [13], [14], [16], [17], [18], [34], [35], [36], [37], [38], [39], [40], [41], [42], [43], [44], [45], [46], [47], [48], [49], [50], [51], [52], [53], [54], [55], [56], [57], [58], [59], [60], [61]. The 8 studies prior to 1940 were experimental and included uncensored and some interval-censored observations. The other 27 studies occurred after 1970 and were all travel-related. These included only interval-censored and right-censored observations related to the period of travel. Altogether there were 131 uncensored observations, 58 interval-censored observations, and 15 right-censored observations. Serotype was not reported for 14 studies, totaling 39 observations (19%). DENV-1 was reported for 102 observations in 12 studies, DENV-2 for 6 observations in 6 studies, DENV-3 for 5 observations in 3 studies, and DENV-4 for 52 observations in 2 studies.

### Extrinsic Incubation Period

**Figure 1:**
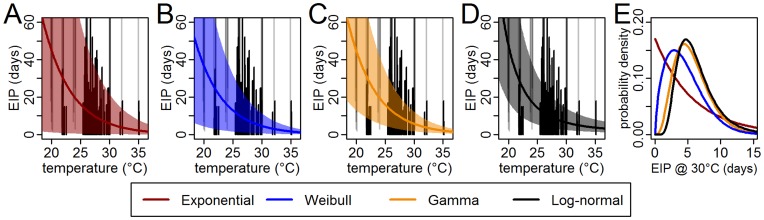
Extrinsic incubation period models. (A–D) Vertical lines indicate the observed censored EIP observations (black for interval-censored and grey for right-censored) at each temperature (with added variability in temperature to improve visualization for observations at the same temperature). Thick solid lines and shaded areas indicate the mean and middle 95%, respectively, of the distribution for each fitted model (red: exponential; blue: Weibull; orange: gamma; and black: log-normal). (E) The lines indicate the predicted probability density for each model at 30°C.

To characterize the EIP, we fitted four time-to-event models with temperature as a covariate and a random effect for each study. The models incorporating temperature and random effects (DIC range: 75–91) fitted better than models without temperature (DIC range: 104–116) and models without random effects (DIC range: 119–129). The 95% credible intervals (CI) for *β_T_* (Table 2) indicate that the association between increased temperature and decreased EIP (Figure 1A–D), is significant for each model. Figure 1 shows the temperature-dependent mean and middle 95% of the respective distributions using the mean parameter estimates (Table 2). Qualitatively, the middle 95% of each estimated distribution crossed through the majority of the observed EIP intervals. The mean estimate for the EIP at 30°C ranged from 4.7 days in the Weibull model to 6.5 days in the log-normal model (Table 2). As measured by DIC, the model with the best fit was the log-normal model, followed by the gamma, Weibull, and exponential models, in order. Due to the close DIC values we performed a bootstrap analysis and found that the log-normal model consistently fit the data better than the other models (Text S3).

**Table 2:** Extrinsic incubation period models.

	ν/τ		β_0_		β_T_		EIP (30°C)		
Model	Mean	95% CI	Mean	95% CI	Mean	95% CI	Mean	95% CI	DIC
Exponential	NA	NA	8	6, 10	−0.20	−0.29, −0.12	6.1	3.4, 9.9	91
Weibull	1.6	1.1, 2.2	−13	−18, −9	0.34	0.21, 0.49	4.7	2.5, 7.3	78
Gamma	4.3	2.5, 6.7	7.9	6.3, 9.7	−0.21	−0.27, −0.14	5.9	3.6, 8.6	76
Log-normal	4.9	2.8, 7.5	2.9	2.3, 3.5	−0.08	−0.10, −0.05	6.5	4.8, 8.8	75
Log-normal†	7	4, 10	1.9	1.2, 2.6	−0.04	−0.069, −0.016	7	5, 10	NA

† without right-censored data, DIC is not comparable as the number of observations is different.

**Figure 2:**
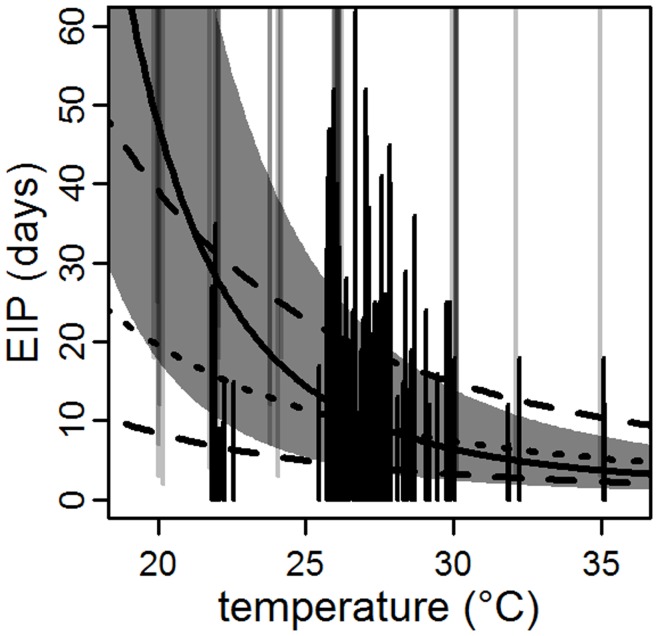
EIP model sensitivity to right-censored observations. The log-normal model is shown as in Figure 1D. The dotted and dashed lines are the comparable predicted mean and middle 95% range, respectively, for the model when the right-censored data is omitted.

Assessment of mosquito infectivity relies on the demonstration of transmission, generally evidenced in this data as dengue in an experimentally exposed individual. Thus, some negative tests of infectivity may be incorrect. We repeated the analysis for the EIP without any of the negative observations which may have resulted from asymptomatic infection. The mean EIP at 30°C was similar to the model with the complete data, but the estimated effect of temperature was reduced (Table 2, Figure 2).

Only three studies contained serotype information for EIP observations, each implicating a single serotype. Because of the limited number of studies, each estimated coefficient was highly correlated with the random effect of the respective study such that inter-study variation could not be separated from potential inter-serotype variation. As only 6 observations were made using *Ae. albopictus*, we did not compare the EIP between species.

Using all of the data and omitting serotype information, the mean estimate for the EIP decreased from 15 days (95% CI: 10, 20 days) at 25°C to 6.5 days (95% CI: 4.8, 8.8 days) at 30°C (Figure 1D) in the log-normal model. To characterize the expected range of EIPs at a given temperature, we estimated the middle 95% of the posterior distribution. At 25°C, the middle 95% of the distribution was from 5 days (95% CI: 3, 8 days) to 33 days (95% CI: 23, 48 days). At 30°C, this range was 2.4 days (95% CI: 1.6, 3.3 days) to 15 days (95% CI: 10, 21 days).

### Intrinsic Incubation Period

Because of the differences between the pre-1940 and post-1970 observations, we modeled these subsets of data as well as the complete dataset independently using each of the 4 models and a random effect for each study. Inclusion of the random effects improved the fit of each model and dataset, with the exception of the exponential model and the post-1970 data (Text S3). The IIP data included multiple observations of each serotype in various studies, but covariates for serotype were not significant and did not improve the fit of the models (Text S3).

**Figure 3:**
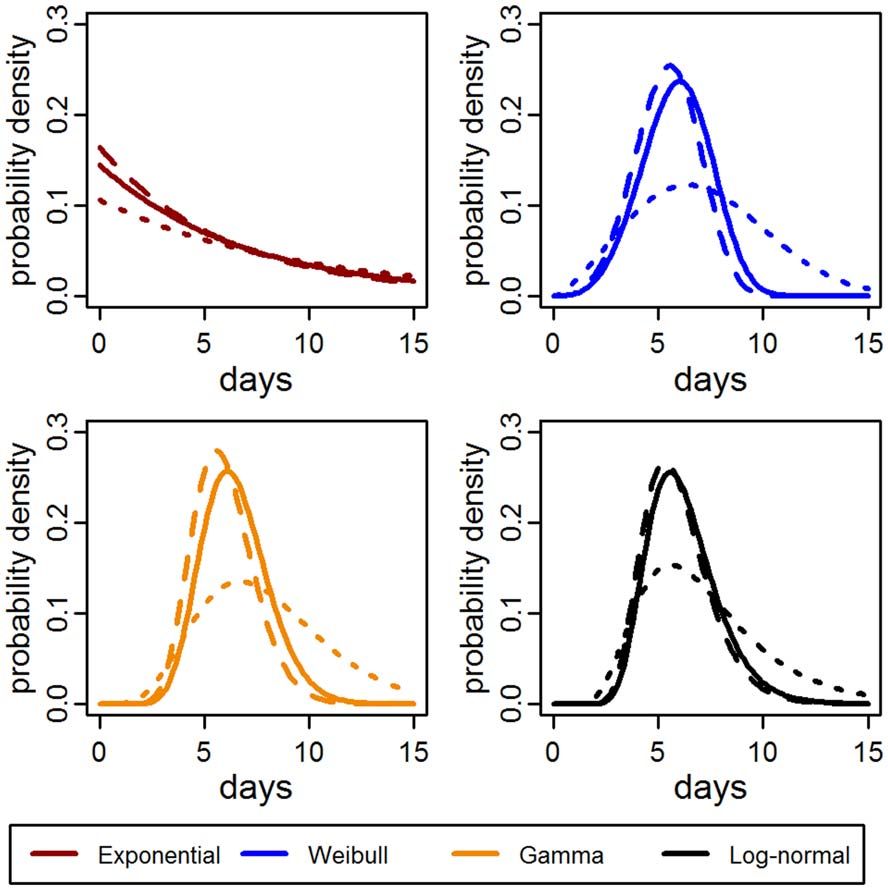
Intrinsic incubation period models and datasets. The thick solid lines indicate the estimated probability distributions using the complete dataset. The dashed and dotted lines indicate the estimate distributions using the pre-1940 and post-1970 subsets, respectively.

As shown in Figure 3, the estimated distributions for the completely censored post-1970 data are flatter than those for the pre-1940 data, likely reflecting the censoring in the observations rather than a change in the IIP. Furthermore, the post-1970 data contributes little to the fit of the complete dataset (Figure 3), so we focus our analysis on the 153 observations from the 8 pre-1940 studies. For this dataset, the gamma model provided the best fit followed by the log-normal, Weibull, and exponential distributions (Table 3). However, for bootstrapped samples of the pre-1940 datasets, the log-normal model provided a better fit in 71 out of 100 samples (Text S3). Thus, while the gamma model may fit the complete dataset better, the log-normal model may better fit any given subset of the data.

**Table 3:** Intrinsic incubation period models fitted with pre-1940s data.

	ν/τ		β_0_		IIP			
Model	Mean	95% CI	Mean	95% CI		Mean	95% CI	DIC
Exponential	–	–	1.8	1.6, 2.0		6.1	4.9, 7.7	768
Weibull	4.0	3.5, 4.4	−7.1	−8.0, −6.2		5.4	4.8, 6.0	476
Gamma	16	13, 20	1.78	1.64, 1.92		5.9	5.2, 6.8	445
Log-normal	13.7	10.9, 16.9	0.56	0.51, 0.60		5.9	5.5, 6.4	460

**Figure 4:**
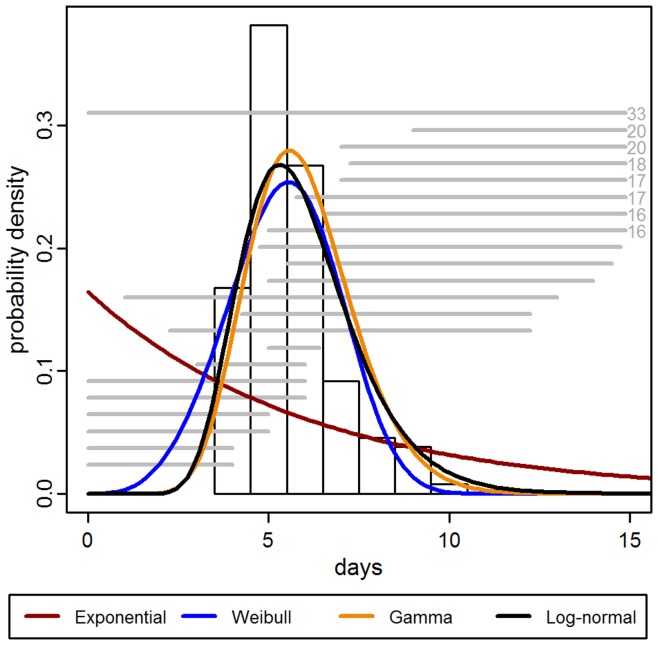
Intrinsic incubation period models. The vertical bars are a histogram of the uncensored IIP data. Horizontal grey lines indicate interval-censored observations from the pre-1940 dataset (those which extend outside of the plot area are labeled with the interval maximum). The curves are the estimated IIPs for each model when fitted to the pre-1940 dataset.

The gamma and log-normal models had similar qualitative fits (Figure 4) and equivalent mean expected IIPs of 5.9 days (Table 3). The middle 95% of the log-normal IIP distribution was 3.4 days (95% CI: 3.0, 3.7 days) to 10 days (95% CI: 9, 11 days). For the gamma distribution, the range was similar, from 3.4 days (95% CI: 2.9, 4.0 days) to 9 days (95% CI: 8, 11 days).

## Discussion

From a total of 38 studies published between 1903 and 2011, we compiled 146 and 204 observations of the EIP and IIP of DENV, respectively. We limited the data to experimental or accidental exposure involving humans, primates (for EIP only), and mosquitoes to better reflect the incubation periods resulting from natural transmission events, rather than highly manipulated experimental ones. Though incubation period determinants may include viral, host, vector, and environmental characteristics [4], [5], [19], [20], [21], [22], [23], [24], [25], [26], there was only sufficient data to assess the role of different serotypes on the EIP and IIP and of mean temperature on the EIP. The other factors, though difficult to measure, are still present and we used random effects to control for inter-study variation associated with the different experimental designs, and mosquito, virus, and human population characteristics present in different studies.

In the analysis of different serotypes, we found no conclusive evidence of differences in EIP or IIP between serotypes. For the IIP, there was a sample of infections due to all four serotypes over a variety of different studies and reports. Controlling for inter-study variation, we found no effect of serotype on IIP. For the EIP, the relevant data was much more limited. With only censored observations, one serotype absent, and the other three represented in single, independent studies, there was not enough information to separate inter-study variation and serotype-associated differences. The difficulty of parsing the effects of distinct genotypes, serotypes, and mosquito populations on the EIP has long been recognized and demonstrated even in highly controlled laboratory studies [22], [24], [62].

The influence of temperature on the EIP of arboviruses has been evident since the early days of arbovirology [63], but here, for the first time, we described it statistically for natural DENV infections. We note, however, that even the temperature dependence as described here is by necessity a simplification of what occurs in the real world. Temperature is constantly varying on spatial and temporal scales and to different extents in different locations. These fluctuations themselves have an influence on the EIP [64]. Furthermore, mosquitoes may modify their exposure to extreme temperatures by spending time inside homes and in shade [65]. Because accounting for all of the subtleties influencing mosquito body temperature is extremely difficult, we used the most general measure readily available, mean temperature. Even with this simplification, we had to estimate the mean temperature for a number of the observations. In spite of this, the temperature-dependence was significant in all models. We further tested this association in the absence of negative infectivity data that could include false-negatives due to asymptomatic infections. While the strength of the temperature association was diminished, it was still statistically significant. Because most of the researchers were exceedingly diligent in monitoring for evidence of infection and negative observations are especially important at lower temperatures when a mosquito may not survive long enough to become infections, we included these observations in the final analysis.

The final models included only the random effects and, for EIP, the mean temperature. Among the EIP models, the log-normal model provided the best fit. For the IIP, the gamma and log-normal models were similar and there was no clearly favored model. We previously found that the yellow fever virus (YFV) EIP data was best described by a Weibull model, with the log-normal model a close second, and that the YFV IIP was best described as by a log-normal distribution [66]. In all cases, the log-normal model provided the optimal or near-optimal fit out of the models investigated. This suggests that the log-normal model may be a good general model for incubation periods for arboviruses, as shown here, as well as for directly transmitted pathogens [67], [68].

The estimated incubation periods described here improve the current understanding of these periods. The DENV EIP is generally referenced as a range of 8–12 days [10], [11]. Here, we found that, given the available data, there is significantly more expected variability, with expected EIP ranges of 5–33 days at 25°C and 2–15 days at 30°C. The IIP range, meanwhile, has been cited as lasting 4–10 days [10] or 3–14 days [11]. Here we estimated that 95% of IIPs are in the 3–10 day range. This range is also in agreement with the previous work of Nishiura and Halstead [15] who fitted log-normal models resulting in a range of approximately 3–9 days. While all of these ranges are similar, our estimates leverage more data from more studies with more diverse vector, human, and virus populations and are based on more flexible models, incorporating covariates, censoring, and different distributional assumptions. These qualities make the estimates more generalizable than those of Nishiura and Halstead and better supported than those generally cited based on observations alone.

## Supporting Information

Text S1 **Data**
(DOCX)

Text S2 **Priors**
(DOCX)

Text S3 **Model Comparisons.**
(DOCX)
