# Supplementary material for: The Incubation Periods of Dengue Viruses
Source: PLoS One. 2012 Nov 30;7(11):e50972. doi: 10.1371/journal.pone.0050972 (PMC3511440; doi:10.1371/journal.pone.0050972)
Supplement: Text S1 — Data (DOCX) [file pone.0050972.s001.docx]

**Text S1: Data**

**Extrinsic Incubation Period Data** (additional notes are below and the full references are in the main manuscript)

| EIP (days) | | Serotype | Temperature (°C) | | | | | | Source | |
| --- | --- | --- | --- | --- | --- | --- | --- | --- | --- | --- |
| Minimum | Maximum |  | Reported mean | Reported minimum | Reported maximum | Time period | Location | Estimated mean | First author | Publication year |
| 0 | 14 | NA | NA | NA | NA | NA | Isthmian Canal Zone, Panama | 28.3 | Carpenter | 1905 |
| 0 | 10 | NA | NA | NA | NA | Jan-May | Brisbane, Australia | 22.4 | Bancroft | 1906 |
| 2 | NA | NA | NA | NA | NA | Apr-May | Sydney, Australia | 20.2 | Cleland | 1916 |
| 0 | 25 | DENV-4 | NA | NA | NA | Sep-Oct | Manila, Philippines | 27.6 | Siler | 1926 |
| 0 | 25 | DENV-4 | NA | NA | NA | Sep-Oct | Manila, Philippines | 27.6 | Siler | 1926 |
| 0 | 14 | DENV-4 | NA | NA | NA | Oct-Nov | Manila, Philippines | 27.2 | Siler | 1926 |
| 0 | 14 | DENV-4 | NA | NA | NA | Oct-Nov | Manila, Philippines | 27.2 | Siler | 1926 |
| 0 | 14 | DENV-4 | NA | NA | NA | Oct-Nov | Manila, Philippines | 27.2 | Siler | 1926 |
| 0 | 16 | DENV-4 | NA | NA | NA | Oct-Nov | Manila, Philippines | 27.2 | Siler | 1926 |
| 9 | 17 | DENV-4 | NA | NA | NA | Oct-Nov | Manila, Philippines | 27.2 | Siler | 1926 |
| 11 | 18 | DENV-4 | NA | NA | NA | Nov | Manila, Philippines | 26.9 | Siler | 1926 |
| 0 | 28 | DENV-4 | NA | NA | NA | Nov-Dec | Manila, Philippines | 26.5 | Siler | 1926 |
| 0 | 28 | DENV-4 | NA | NA | NA | Nov-Dec | Manila, Philippines | 26.5 | Siler | 1926 |
| 0 | 12 | DENV-4 | NA | NA | NA | Nov | Manila, Philippines | 26.9 | Siler | 1926 |
| 0 | 11 | DENV-4 | NA | NA | NA | Nov | Manila, Philippines | 26.9 | Siler | 1926 |
| 10 | 20 | DENV-4 | NA | NA | NA | Nov-Dec | Manila, Philippines | 26.5 | Siler | 1926 |
| 0 | 27 | DENV-4 | NA | NA | NA | Dec | Manila, Philippines | 26.1 | Siler | 1926 |
| 0 | 20 | DENV-4 | NA | NA | NA | Dec | Manila, Philippines | 26.1 | Siler | 1926 |
| 0 | 35 | DENV-4 | NA | NA | NA | Dec-Jan | Manila, Philippines | 25.9 | Siler | 1926 |
| 0 | 32 | DENV-4 | NA | NA | NA | Dec-Jan | Manila, Philippines | 25.9 | Siler | 1926 |
| 0 | 20 | DENV-4 | NA | NA | NA | Dec | Manila, Philippines | 26.1 | Siler | 1926 |
| 0 | 28 | DENV-4 | NA | NA | NA | Dec-Jan | Manila, Philippines | 25.9 | Siler | 1926 |
| 0 | 16 | DENV-4 | NA | NA | NA | Dec-Jan | Manila, Philippines | 25.9 | Siler | 1926 |
| 0 | 31 | DENV-4 | NA | NA | NA | Jan-Feb | Manila, Philippines | 26.0 | Siler | 1926 |
| 0 | 31 | DENV-4 | NA | NA | NA | Jan-Feb | Manila, Philippines | 26.0 | Siler | 1926 |
| 8 | 19 | DENV-4 | NA | NA | NA | Jan-Feb | Manila, Philippines | 26.0 | Siler | 1926 |
| 9 | 19 | DENV-4 | NA | NA | NA | Jan-Feb | Manila, Philippines | 26.0 | Siler | 1926 |
| 10 | 19 | DENV-4 | NA | NA | NA | Jan-Feb | Manila, Philippines | 26.0 | Siler | 1926 |
| 0 | 18 | DENV-4 | NA | NA | NA | Jan-Feb | Manila, Philippines | 26.0 | Siler | 1926 |
| 0 | 14 | DENV-4 | NA | NA | NA | Feb | Manila, Philippines | 26.2 | Siler | 1926 |
| 0 | 18 | DENV-4 | NA | NA | NA | Feb | Manila, Philippines | 26.2 | Siler | 1926 |
| 0 | 15 | DENV-4 | NA | NA | NA | Feb | Manila, Philippines | 26.2 | Siler | 1926 |
| 0 | 21 | DENV-4 | NA | NA | NA | Feb-Mar | Manila, Philippines | 27.0 | Siler | 1926 |
| 0 | 21 | DENV-4 | NA | NA | NA | Feb-Mar | Manila, Philippines | 27.0 | Siler | 1926 |
| 0 | 62 | DENV-4 | NA | NA | NA | Jan-Mar | Manila, Philippines | 26.5 | Siler | 1926 |
| 0 | 13 | NA | NA | 21.5 | 33.4 | NA | Fort Mills, Philippines | NA | Schule | 1928 |
| 0 | 13 | NA | NA | 21.5 | 33.4 | NA | Fort Mills, Philippines | NA | Schule | 1928 |
| 0 | 14 | NA | NA | 21.5 | 33.4 | NA | Fort Mills, Philippines | NA | Schule | 1928 |
| 0 | 14 | NA | NA | 21.5 | 33.4 | NA | Fort Mills, Philippines | NA | Schule | 1928 |
| 0 | 16 | NA | NA | 21.5 | 33.4 | NA | Fort Mills, Philippines | NA | Schule | 1928 |
| 0 | 8 | NA | NA | 21.5 | 33.4 | NA | Fort Mills, Philippines | NA | Schule | 1928 |
| 0 | 10 | NA | NA | 21.5 | 33.4 | NA | Fort Mills, Philippines | NA | Schule | 1928 |
| 0 | 25 | NA | NA | 21.5 | 33.4 | NA | Fort Mills, Philippines | NA | Schule | 1928 |
| 4 | NA | NA | 22 | NA | NA | NA | Athens, Greece | NA | Blanc | 1930 |
| 0 | 9 | NA | 22 | NA | NA | NA | Athens, Greece | NA | Blanc | 1930 |
| 0 | 13 | NA | 22 | NA | NA | NA | Athens, Greece | NA | Blanc | 1930 |
| 18 | NA | NA | 22 | NA | NA | NA | Athens, Greece | NA | Blanc | 1930 |
| 0 | 27 | NA | 22 | NA | NA | NA | Athens, Greece | NA | Blanc | 1930 |
| 4 | NA | NA | 22 | NA | NA | NA | Athens, Greece | NA | Blanc | 1930 |
| 0 | 9 | NA | 22 | NA | NA | NA | Athens, Greece | NA | Blanc | 1930 |
| 67 | NA | NA | 16.4 | NA | NA | NA | Athens, Greece | NA | Blanc | 1930 |
| 22 | NA | NA | 22 | NA | NA | NA | Athens, Greece | NA | Blanc | 1930 |
| 0 | 35 | NA | 22 | NA | NA | NA | Athens, Greece | NA | Blanc | 1930 |
| 23 | NA | NA | 22 | NA | NA | NA | Athens, Greece | NA | Blanc | 1930 |
| 0 | 29 | NA | 22 | NA | NA | NA | Athens, Greece | NA | Blanc | 1930 |
| 79 | NA | NA | 16.4 | NA | NA | NA | Athens, Greece | NA | Blanc | 1930 |
| 91 | NA | NA | 16.4 | NA | NA | NA | Athens, Greece | NA | Blanc | 1930 |
| 0 | 15 | NA | 22.5 | NA | NA | NA | Athens, Greece | NA | Blanc | 1930 |
| 0 | 15 | NA | 22.5 | NA | NA | NA | Athens, Greece | NA | Blanc | 1930 |
| 0 | 16 | DENV-1 | NA | NA | NA | Dec | Manila, Philippines | 26.1 | Simmons | 1931 |
| 0 | 41 | DENV-1 | NA | NA | NA | Jan-Feb | Manila, Philippines | 26.0 | Simmons | 1931 |
| 0 | 40 | DENV-1 | NA | NA | NA | Jan-Feb | Manila, Philippines | 26.0 | Simmons | 1931 |
| 0 | 45 | DENV-1 | NA | NA | NA | Feb-Apr | Manila, Philippines | 27.7 | Simmons | 1931 |
| 0 | 40 | DENV-1 | NA | NA | NA | Feb-Apr | Manila, Philippines | 27.7 | Simmons | 1931 |
| 0 | 24 | DENV-1 | NA | NA | NA | Apr-May | Manila, Philippines | 29.3 | Simmons | 1931 |
| 0 | 12 | DENV-1 | NA | NA | NA | Apr-May | Manila, Philippines | 29.3 | Simmons | 1931 |
| 0 | 16 | DENV-1 | NA | NA | NA | May | Manila, Philippines | 29.4 | Simmons | 1931 |
| 0 | 36 | DENV-1 | NA | NA | NA | May-Jun | Manila, Philippines | 28.9 | Simmons | 1931 |
| 0 | 15 | DENV-1 | NA | NA | NA | Jun-Jul | Manila, Philippines | 28.2 | Simmons | 1931 |
| 0 | 13 | DENV-1 | NA | NA | NA | Jul | Manila, Philippines | 27.9 | Simmons | 1931 |
| 0 | 13 | DENV-1 | NA | NA | NA | Jul | Manila, Philippines | 27.9 | Simmons | 1931 |
| 0 | 12 | DENV-1 | NA | NA | NA | Jul | Manila, Philippines | 27.9 | Simmons | 1931 |
| 0 | 22 | DENV-1 | NA | NA | NA | Aug | Manila, Philippines | 27.4 | Simmons | 1931 |
| 0 | 20 | DENV-1 | NA | NA | NA | Aug | Manila, Philippines | 27.4 | Simmons | 1931 |
| 0 | 15 | DENV-1 | NA | NA | NA | Aug | Manila, Philippines | 27.4 | Simmons | 1931 |
| 0 | 19 | DENV-1 | NA | NA | NA | Aug-Sep | Manila, Philippines | 27.5 | Simmons | 1931 |
| 0 | 41 | DENV-1 | NA | NA | NA | Aug-Oct | Manila, Philippines | 27.5 | Simmons | 1931 |
| 0 | 15 | DENV-1 | NA | NA | NA | Aug-Sep | Manila, Philippines | 27.5 | Simmons | 1931 |
| 0 | 26 | DENV-1 | NA | NA | NA | Sep-Oct | Manila, Philippines | 27.6 | Simmons | 1931 |
| 0 | 25 | DENV-1 | NA | NA | NA | Sep-Oct | Manila, Philippines | 27.6 | Simmons | 1931 |
| 0 | 14 | DENV-1 | NA | NA | NA | Oct | Manila, Philippines | 27.5 | Simmons | 1931 |
| 0 | 21 | DENV-1 | NA | NA | NA | Aug | Manila, Philippines | 27.4 | Simmons | 1931 |
| 0 | 19 | DENV-1 | NA | NA | NA | Oct-Nov | Manila, Philippines | 27.2 | Simmons | 1931 |
| 0 | 23 | DENV-1 | NA | NA | NA | Nov | Manila, Philippines | 26.9 | Simmons | 1931 |
| 0 | 20 | DENV-1 | NA | NA | NA | Nov | Manila, Philippines | 26.9 | Simmons | 1931 |
| 0 | 27 | DENV-1 | NA | NA | NA | Nov-Dec | Manila, Philippines | 26.5 | Simmons | 1931 |
| 0 | 24 | DENV-1 | NA | NA | NA | Nov-Dec | Manila, Philippines | 26.5 | Simmons | 1931 |
| 0 | 14 | DENV-1 | NA | NA | NA | Nov-Dec | Manila, Philippines | 26.5 | Simmons | 1931 |
| 0 | 17 | DENV-1 | NA | NA | NA | Nov-Dec | Manila, Philippines | 26.5 | Simmons | 1931 |
| 0 | 52 | DENV-1 | NA | NA | NA | Dec-Feb | Manila, Philippines | 26.0 | Simmons | 1931 |
| 0 | 33 | DENV-1 | NA | NA | NA | Dec-Jan | Manila, Philippines | 25.9 | Simmons | 1931 |
| 0 | 17 | DENV-1 | NA | NA | NA | Jan | Manila, Philippines | 25.7 | Simmons | 1931 |
| 0 | 32 | DENV-1 | NA | NA | NA | Dec-Jan | Manila, Philippines | 25.9 | Simmons | 1931 |
| 0 | 47 | DENV-1 | NA | NA | NA | Dec-Feb | Manila, Philippines | 26.0 | Simmons | 1931 |
| 0 | 41 | DENV-1 | NA | NA | NA | Dec-Jan | Manila, Philippines | 25.9 | Simmons | 1931 |
| 0 | 26 | DENV-1 | NA | NA | NA | Jan-Feb | Manila, Philippines | 26.0 | Simmons | 1931 |
| 0 | 26 | DENV-1 | NA | NA | NA | Jan-Feb | Manila, Philippines | 26.0 | Simmons | 1931 |
| 0 | 22 | DENV-1 | NA | NA | NA | Feb | Manila, Philippines | 26.2 | Simmons | 1931 |
| 0 | 15 | DENV-1 | NA | NA | NA | Feb | Manila, Philippines | 26.2 | Simmons | 1931 |
| 0 | 52 | DENV-1 | NA | NA | NA | Feb-Mar | Manila, Philippines | 27.0 | Simmons | 1931 |
| 0 | 46 | DENV-1 | NA | NA | NA | Feb-Mar | Manila, Philippines | 27.0 | Simmons | 1931 |
| 0 | 19 | DENV-1 | NA | NA | NA | Feb-Mar | Manila, Philippines | 27.0 | Simmons | 1931 |
| 0 | 19 | DENV-1 | NA | NA | NA | Feb-Mar | Manila, Philippines | 27.0 | Simmons | 1931 |
| 0 | 37 | DENV-1 | NA | NA | NA | Feb-Mar | Manila, Philippines | 27.0 | Simmons | 1931 |
| 0 | 29 | DENV-1 | NA | NA | NA | Mar | Manila, Philippines | 27.7 | Simmons | 1931 |
| 0 | 29 | DENV-1 | NA | NA | NA | Mar-Apr | Manila, Philippines | 28.5 | Simmons | 1931 |
| 0 | 19 | DENV-1 | NA | NA | NA | Mar-Apr | Manila, Philippines | 28.5 | Simmons | 1931 |
| 0 | 18 | DENV-1 | NA | NA | NA | Oct | Manila, Philippines | 27.5 | Simmons | 1931 |
| 3 | NA | DENV-2 | 20 | NA | NA | NA | Bangkok, Thailand | NA | Watts | 1987 |
| 7 | NA | DENV-2 | 20 | NA | NA | NA | Bangkok, Thailand | NA | Watts | 1987 |
| 12 | NA | DENV-2 | 20 | NA | NA | NA | Bangkok, Thailand | NA | Watts | 1987 |
| 18 | NA | DENV-2 | 20 | NA | NA | NA | Bangkok, Thailand | NA | Watts | 1987 |
| 25 | NA | DENV-2 | 20 | NA | NA | NA | Bangkok, Thailand | NA | Watts | 1987 |
| 3 | NA | DENV-2 | 24 | NA | NA | NA | Bangkok, Thailand | NA | Watts | 1987 |
| 7 | NA | DENV-2 | 24 | NA | NA | NA | Bangkok, Thailand | NA | Watts | 1987 |
| 12 | NA | DENV-2 | 24 | NA | NA | NA | Bangkok, Thailand | NA | Watts | 1987 |
| 18 | NA | DENV-2 | 24 | NA | NA | NA | Bangkok, Thailand | NA | Watts | 1987 |
| 25 | NA | DENV-2 | 24 | NA | NA | NA | Bangkok, Thailand | NA | Watts | 1987 |
| 3 | NA | DENV-2 | 26 | NA | NA | NA | Bangkok, Thailand | NA | Watts | 1987 |
| 7 | NA | DENV-2 | 26 | NA | NA | NA | Bangkok, Thailand | NA | Watts | 1987 |
| 12 | NA | DENV-2 | 26 | NA | NA | NA | Bangkok, Thailand | NA | Watts | 1987 |
| 18 | NA | DENV-2 | 26 | NA | NA | NA | Bangkok, Thailand | NA | Watts | 1987 |
| 25 | NA | DENV-2 | 26 | NA | NA | NA | Bangkok, Thailand | NA | Watts | 1987 |
| 3 | NA | DENV-2 | 30 | NA | NA | NA | Bangkok, Thailand | NA | Watts | 1987 |
| 7 | NA | DENV-2 | 30 | NA | NA | NA | Bangkok, Thailand | NA | Watts | 1987 |
| 12 | NA | DENV-2 | 30 | NA | NA | NA | Bangkok, Thailand | NA | Watts | 1987 |
| 18 | NA | DENV-2 | 30 | NA | NA | NA | Bangkok, Thailand | NA | Watts | 1987 |
| 0 | 25 | DENV-2 | 30 | NA | NA | NA | Bangkok, Thailand | NA | Watts | 1987 |
| 3 | NA | DENV-2 | 26 | NA | NA | NA | Bangkok, Thailand | NA | Watts | 1987 |
| 7 | NA | DENV-2 | 26 | NA | NA | NA | Bangkok, Thailand | NA | Watts | 1987 |
| 12 | NA | DENV-2 | 26 | NA | NA | NA | Bangkok, Thailand | NA | Watts | 1987 |
| 18 | NA | DENV-2 | 26 | NA | NA | NA | Bangkok, Thailand | NA | Watts | 1987 |
| 25 | NA | DENV-2 | 26 | NA | NA | NA | Bangkok, Thailand | NA | Watts | 1987 |
| 3 | NA | DENV-2 | 30 | NA | NA | NA | Bangkok, Thailand | NA | Watts | 1987 |
| 7 | NA | DENV-2 | 30 | NA | NA | NA | Bangkok, Thailand | NA | Watts | 1987 |
| 0 | 12 | DENV-2 | 30 | NA | NA | NA | Bangkok, Thailand | NA | Watts | 1987 |
| 0 | 18 | DENV-2 | 30 | NA | NA | NA | Bangkok, Thailand | NA | Watts | 1987 |
| 0 | 25 | DENV-2 | 30 | NA | NA | NA | Bangkok, Thailand | NA | Watts | 1987 |
| 3 | NA | DENV-2 | 32 | NA | NA | NA | Bangkok, Thailand | NA | Watts | 1987 |
| 0 | 7 | DENV-2 | 32 | NA | NA | NA | Bangkok, Thailand | NA | Watts | 1987 |
| 0 | 12 | DENV-2 | 32 | NA | NA | NA | Bangkok, Thailand | NA | Watts | 1987 |
| 0 | 18 | DENV-2 | 32 | NA | NA | NA | Bangkok, Thailand | NA | Watts | 1987 |
| 3 | NA | DENV-2 | 35 | NA | NA | NA | Bangkok, Thailand | NA | Watts | 1987 |
| 0 | 7 | DENV-2 | 35 | NA | NA | NA | Bangkok, Thailand | NA | Watts | 1987 |
| 0 | 12 | DENV-2 | 35 | NA | NA | NA | Bangkok, Thailand | NA | Watts | 1987 |
| 0 | 18 | DENV-2 | 35 | NA | NA | NA | Bangkok, Thailand | NA | Watts | 1987 |

**Additional notes regarding data selection from selected articles:**

Watts et al., 1987. There are two experiments reported. In the first experiment no viremia was detected in the monkey that provided the original infectious blood meal for the mosquitoes. Despite this, that blood meal did prove infectious for a number of mosquitoes and seroconversion occurred in the monkey. However, only a single pool of mosquitoes was shown to be infectious (the 30**°**C pool after 25 days). The second experiment, meanwhile, used a demonstrably viremic monkey and produced much higher rates of infection in the mosquitoes and subsequent transmission. Because of the substantial known difference between the two experiments, we assigned each a unique random effect. For both experiments, we treated a pool of mosquitoes that did not transmit the virus as a single, right-censored observation. We record it as a single observation because the exact number of mosquitoes that fed at each time point is not reported (the reported range is 42-100%). Any pool that successfully transmitted the virus is treated as a single interval-censored observation, as it is clear that at least one mosquito was infectious.

Schule, 1928. Schule decided that if 9-10 days passed without evidence of disease that an individual had not been successfully infected and then used for a subsequent experiment. Here, we conservatively assume that an initial exposure could cause illness even after this time period. Thus, instead of assuming that the first experiment represents a negative assessment of mosquito infectivity, we assume that the infectivity status is unknown at that time. Thus all of the observations are treated as interval-censored from zero to the last potential day of exposure prior to the onset illness.

Blanc and Caminopetros, 1930. The authors carried out a number of infectivity experiments with pools of mosquitoes, exposing multiple volunteers to the pools over periods of several months. In captivity, mosquitoes were able to feed on raisins and, occasionally, on DENV-immune humans. Because of these alternate food sources and the absence of a feeding record, it is unknown whether each mosquito fed during each transmission experiment. We therefore assume that initial negative feedings represent right-censored observations, i.e. that at least one feeding mosquito had been infected but had not yet become infectious. And we assume that the first positive feeding represents a censored interval from the earliest potential time of infection to the time of feeding. For the wild caught mosquitoes that were not experimentally exposed, we know nothing of the EIP because some may have been infectious before capture and may not have fed at early time points in captivity. Lastly, observations for pools of mosquitoes that were maintained at different temperatures for different time periods were only used from the time period corresponding to the initial temperature.

Simmons, St. John, and Reynolds, 1931. Most of the EIP observations were recorded in Table 14 though the text was also reviewed for additional information. In the case of disagreement between the table and the text descriptions, we used the descriptions as they contain more detail.

**Intrinsic Incubation Period Data**

| IIP (days) | | | Serotype | Source | |
| --- | --- | --- | --- | --- | --- |
| Actual | Minimum | Maximum |  | First author | Publication year |
| NA | 0 | 5 | NA | Graham | 1903 |
| NA | 0 | 6 | NA | Graham | 1903 |
| NA | 0 | 4 | NA | Graham | 1903 |
| NA | 0 | 4 | NA | Graham | 1903 |
| NA | 0 | 5 | NA | Graham | 1903 |
| NA | 0 | 6 | NA | Graham | 1903 |
| 6 | NA | NA | NA | Bancroft | 1906 |
| 5 | NA | NA | NA | Bancroft | 1906 |
| 8.2 | NA | NA | NA | Cleland | 1916 |
| NA | 5 | 6.4 | NA | Cleland | 1916 |
| 9.4 | NA | NA | NA | Cleland | 1916 |
| 7.8 | NA | NA | NA | Cleland | 1916 |
| 5.8 | NA | NA | NA | Chandler | 1923 |
| 6.2 | NA | NA | NA | Chandler | 1923 |
| 6.5 | NA | NA | NA | Chandler | 1923 |
| 4.1 | NA | NA | NA | Chandler | 1923 |
| NA | 0 | 33 | NA | Chandler | 1923 |
| 4.25 | NA | NA | DENV-4 | Siler | 1926 |
| 4.5 | NA | NA | DENV-4 | Siler | 1926 |
| 5.5 | NA | NA | DENV-4 | Siler | 1926 |
| 5.75 | NA | NA | DENV-4 | Siler | 1926 |
| 5.5 | NA | NA | DENV-4 | Siler | 1926 |
| 7.25 | NA | NA | DENV-4 | Siler | 1926 |
| 7.5 | NA | NA | DENV-4 | Siler | 1926 |
| 5.75 | NA | NA | DENV-4 | Siler | 1926 |
| 4.5 | NA | NA | DENV-4 | Siler | 1926 |
| 4.75 | NA | NA | DENV-4 | Siler | 1926 |
| 9.25 | NA | NA | DENV-4 | Siler | 1926 |
| NA | 4.25 | 12.25 | DENV-4 | Siler | 1926 |
| 8.25 | NA | NA | DENV-4 | Siler | 1926 |
| 6.75 | NA | NA | DENV-4 | Siler | 1926 |
| NA | 7.25 | 18.25 | DENV-4 | Siler | 1926 |
| 4.75 | NA | NA | DENV-4 | Siler | 1926 |
| 8.5 | NA | NA | DENV-4 | Siler | 1926 |
| 5.25 | NA | NA | DENV-4 | Siler | 1926 |
| NA | 4.75 | 14.75 | DENV-4 | Siler | 1926 |
| 10 | NA | NA | DENV-4 | Siler | 1926 |
| 8.25 | NA | NA | DENV-4 | Siler | 1926 |
| 6.75 | NA | NA | DENV-4 | Siler | 1926 |
| 7.75 | NA | NA | DENV-4 | Siler | 1926 |
| 4.25 | NA | NA | DENV-4 | Siler | 1926 |
| 6.25 | NA | NA | DENV-4 | Siler | 1926 |
| 5.25 | NA | NA | DENV-4 | Siler | 1926 |
| 4.25 | NA | NA | DENV-4 | Siler | 1926 |
| 5.25 | NA | NA | DENV-4 | Siler | 1926 |
| 5.5 | NA | NA | DENV-4 | Siler | 1926 |
| 5.5 | NA | NA | DENV-4 | Siler | 1926 |
| 5.5 | NA | NA | DENV-4 | Siler | 1926 |
| 5.75 | NA | NA | DENV-4 | Siler | 1926 |
| NA | 5.75 | 17 | DENV-4 | Siler | 1926 |
| NA | 6.5 | 16.5 | DENV-4 | Siler | 1926 |
| NA | 5.5 | 14.5 | DENV-4 | Siler | 1926 |
| 5.25 | NA | NA | DENV-4 | Siler | 1926 |
| 5.25 | NA | NA | DENV-4 | Siler | 1926 |
| 7.5 | NA | NA | DENV-4 | Siler | 1926 |
| 5.5 | NA | NA | DENV-4 | Siler | 1926 |
| 7.5 | NA | NA | DENV-4 | Siler | 1926 |
| 6.25 | NA | NA | DENV-4 | Siler | 1926 |
| 6.75 | NA | NA | DENV-4 | Siler | 1926 |
| 5 | NA | NA | DENV-4 | Siler | 1926 |
| 6.5 | NA | NA | DENV-4 | Siler | 1926 |
| 4.75 | NA | NA | DENV-4 | Siler | 1926 |
| 4.75 | NA | NA | DENV-4 | Siler | 1926 |
| 4.5 | NA | NA | DENV-4 | Siler | 1926 |
| 6 | NA | NA | NA | Schule | 1928 |
| NA | 7 | 20 | NA | Schule | 1928 |
| NA | 5 | 16 | NA | Schule | 1928 |
| NA | 9 | 20 | NA | Schule | 1928 |
| NA | 5 | 14 | NA | Schule | 1928 |
| NA | 7 | 17 | NA | Schule | 1928 |
| 9 | NA | NA | NA | Schule | 1928 |
| 6 | NA | NA | NA | Schule | 1928 |
| 5 | NA | NA | NA | Schule | 1928 |
| 6 | NA | NA | NA | Snijders | 1931 |
| NA | 3 | 6 | NA | Snijders | 1931 |
| 5 | NA | NA | NA | Snijders | 1931 |
| 9 | NA | NA | DENV-1 | Simmons | 1931 |
| 7 | NA | NA | DENV-1 | Simmons | 1931 |
| 6 | NA | NA | DENV-1 | Simmons | 1931 |
| 7.5 | NA | NA | DENV-1 | Simmons | 1931 |
| 5 | NA | NA | DENV-1 | Simmons | 1931 |
| 5 | NA | NA | DENV-1 | Simmons | 1931 |
| 5 | NA | NA | DENV-1 | Simmons | 1931 |
| 6 | NA | NA | DENV-1 | Simmons | 1931 |
| 6 | NA | NA | DENV-1 | Simmons | 1931 |
| 4.5 | NA | NA | DENV-1 | Simmons | 1931 |
| 5.5 | NA | NA | DENV-1 | Simmons | 1931 |
| 6 | NA | NA | DENV-1 | Simmons | 1931 |
| 6 | NA | NA | DENV-1 | Simmons | 1931 |
| 4.5 | NA | NA | DENV-1 | Simmons | 1931 |
| 7 | NA | NA | DENV-1 | Simmons | 1931 |
| 5.5 | NA | NA | DENV-1 | Simmons | 1931 |
| 6.25 | NA | NA | DENV-1 | Simmons | 1931 |
| 4.5 | NA | NA | DENV-1 | Simmons | 1931 |
| 5 | NA | NA | DENV-1 | Simmons | 1931 |
| 6 | NA | NA | DENV-1 | Simmons | 1931 |
| 4.25 | NA | NA | DENV-1 | Simmons | 1931 |
| 4 | NA | NA | DENV-1 | Simmons | 1931 |
| 5 | NA | NA | DENV-1 | Simmons | 1931 |
| 4.5 | NA | NA | DENV-1 | Simmons | 1931 |
| 6 | NA | NA | DENV-1 | Simmons | 1931 |
| 6.5 | NA | NA | DENV-1 | Simmons | 1931 |
| 5 | NA | NA | DENV-1 | Simmons | 1931 |
| 7 | NA | NA | DENV-1 | Simmons | 1931 |
| 5 | NA | NA | DENV-1 | Simmons | 1931 |
| 6 | NA | NA | DENV-1 | Simmons | 1931 |
| 4.25 | NA | NA | DENV-1 | Simmons | 1931 |
| 5.5 | NA | NA | DENV-1 | Simmons | 1931 |
| 4.25 | NA | NA | DENV-1 | Simmons | 1931 |
| 5 | NA | NA | DENV-1 | Simmons | 1931 |
| 5 | NA | NA | DENV-1 | Simmons | 1931 |
| 5.5 | NA | NA | DENV-1 | Simmons | 1931 |
| 5 | NA | NA | DENV-1 | Simmons | 1931 |
| 5 | NA | NA | DENV-1 | Simmons | 1931 |
| 6.5 | NA | NA | DENV-1 | Simmons | 1931 |
| 5.5 | NA | NA | DENV-1 | Simmons | 1931 |
| NA | 1 | 13 | DENV-1 | Simmons | 1931 |
| 6 | NA | NA | DENV-1 | Simmons | 1931 |
| 5 | NA | NA | DENV-1 | Simmons | 1931 |
| 7 | NA | NA | DENV-1 | Simmons | 1931 |
| 5 | NA | NA | DENV-1 | Simmons | 1931 |
| 6 | NA | NA | DENV-1 | Simmons | 1931 |
| 5 | NA | NA | DENV-1 | Simmons | 1931 |
| 4.5 | NA | NA | DENV-1 | Simmons | 1931 |
| 6 | NA | NA | DENV-1 | Simmons | 1931 |
| 6 | NA | NA | DENV-1 | Simmons | 1931 |
| 5 | NA | NA | DENV-1 | Simmons | 1931 |
| 5 | NA | NA | DENV-1 | Simmons | 1931 |
| 6.5 | NA | NA | DENV-1 | Simmons | 1931 |
| 5 | NA | NA | DENV-1 | Simmons | 1931 |
| 5 | NA | NA | DENV-1 | Simmons | 1931 |
| 6 | NA | NA | DENV-1 | Simmons | 1931 |
| 4 | NA | NA | DENV-1 | Simmons | 1931 |
| 5.5 | NA | NA | DENV-1 | Simmons | 1931 |
| 5 | NA | NA | DENV-1 | Simmons | 1931 |
| 6 | NA | NA | DENV-1 | Simmons | 1931 |
| 4.5 | NA | NA | DENV-1 | Simmons | 1931 |
| 4.5 | NA | NA | DENV-1 | Simmons | 1931 |
| 5 | NA | NA | DENV-1 | Simmons | 1931 |
| 6 | NA | NA | DENV-1 | Simmons | 1931 |
| 5.5 | NA | NA | DENV-1 | Simmons | 1931 |
| 4.5 | NA | NA | DENV-1 | Simmons | 1931 |
| 5 | NA | NA | DENV-1 | Simmons | 1931 |
| 4.5 | NA | NA | DENV-1 | Simmons | 1931 |
| 6.25 | NA | NA | DENV-1 | Simmons | 1931 |
| 6.25 | NA | NA | DENV-1 | Simmons | 1931 |
| 5 | NA | NA | DENV-1 | Simmons | 1931 |
| 6.25 | NA | NA | DENV-1 | Simmons | 1931 |
| 4.5 | NA | NA | DENV-1 | Simmons | 1931 |
| 5.25 | NA | NA | DENV-1 | Simmons | 1931 |
| NA | 2.25 | 12.25 | DENV-1 | Simmons | 1931 |
| 9.25 | NA | NA | DENV-1 | Simmons | 1931 |
| 5 | NA | NA | DENV-1 | Simmons | 1931 |
| NA | 2 | 44 | DENV-2 | Iwamoto | 1973 |
| NA | 1 | 37 | DENV-2 | Schleupner | 1977 |
| NA | 0 | 6 | DENV-4 | CDC | 1982 |
| NA | 5 | 11 | DENV-4 | CDC | 1982 |
| NA | 5 | 11 | DENV-4 | CDC | 1982 |
| NA | 5 | 11 | DENV-4 | CDC | 1982 |
| NA | 5 | 11 | DENV-4 | CDC | 1982 |
| NA | 5 | 22 | DENV-1 | Malison | 1983 |
| NA | 6 | 23 | DENV-1 | Malison | 1983 |
| NA | 0 | 123 | DENV-1 | Malison | 1983 |
| NA | 5 | 12 | DENV-1 | Malison | 1983 |
| NA | 0 | 129 | DENV-1 | Malison | 1983 |
| NA | 7 | NA | DENV-1 | San Juan Laboratories | 1986 |
| NA | 7 | NA | DENV-1 | Blackburn | 1987 |
| NA | 0 | 61 | NA | Jacobs | 1991 |
| NA | 0 | 14 | NA | Jacobs | 1991 |
| NA | 6 | NA | DENV-3 | Melissant | 1992 |
| NA | 0 | 58 | DENV-1 | Patey | 1993 |
| NA | 2 | 30 | NA | Patey | 1993 |
| NA | 3 | 8 | NA | McCarthy | 1995 |
| NA | 1 | 18 | DENV-1 | Amendola | 1996 |
| NA | 0 | 17 | DENV-1 | Amendola | 1996 |
| NA | 2 | 22 | DENV-2 | Teichmann | 1998 |
| NA | 0 | 70 | DENV-1 | Durand | 2000 |
| NA | 3 | NA | NA | Helbok | 2004 |
| NA | 0 | 31 | NA | Helbok | 2004 |
| NA | 4 | NA | NA | Nilsson | 2005 |
| NA | 7 | NA | NA | Gasperino | 2007 |
| NA | 6 | 26 | DENV-2 | Jensenius | 2007 |
| NA | 5 | NA | DENV-1 | Tavakoli | 2007 |
| NA | 0 | 4 | DENV-3 | Morita | 2008 |
| NA | 0 | 21 | DENV-2 | Takasaki | 2008 |
| NA | 0 | 27 | DENV-2 | Cardosa | 2009 |
| NA | 0 | 9 | NA | Courtney | 2009 |
| NA | 3 | NA | NA | Ninove | 2009 |
| NA | 0 | 7 | DENV-1 | CDC | 2010 |
| NA | 4 | NA | DENV-1 | Vainio | 2010 |
| NA | 1 | NA | DENV-3 | Vainio | 2010 |
| NA | 2 | NA | DENV-1 | Vainio | 2010 |
| NA | 3 | NA | DENV-3 | Vainio | 2010 |
| NA | 2 | NA | DENV-1 | Vainio | 2010 |
| NA | 1 | NA | DENV-3 | Vainio | 2010 |
| NA | 1 | NA | DENV-1 | Vainio | 2010 |
| NA | 3 | 24 | DENV-1 | Waagsbo | 2010 |
| NA | 3 | 10 | DENV-1 | Anderson | 2011 |
| NA | 3 | 10 | DENV-1 | Anderson | 2011 |
| NA | 4 | 11 | DENV-1 | Anderson | 2011 |
| NA | 4 | 11 | DENV-1 | Anderson | 2011 |
| NA | 6 | 13 | DENV-1 | Anderson | 2011 |
| NA | 6 | 13 | DENV-1 | Anderson | 2011 |
| NA | 7 | 14 | DENV-1 | Anderson | 2011 |

**Additional notes regarding data selection from selected articles:**

Simmons, St. John, and Reynolds, 1931. All of the experiments on American volunteers resulting in IIP observations are reported in Table 14. We used this table as the basis for data selection, as it has more IIP observations than are recorded in the experiment descriptions in the rest of the text. However, we also reviewed the text to corroborate that data and identify other important considerations. For example, many volunteers were exposed to DENV via non-natural or now discredited (thanks to this work) transmission mechanisms such as feeding by *Culex* mosquitoes, grinding infectious mosquitoes into abraded skin, or inoculating volunteers with infectious blood. All experiments in which feeding by naturally infected *Aedes* was not the source of infection were discarded. Some of the volunteers used in these unsuccessful experiments were used for later successful experiments. In these cases, as long as the early exposure was via an unlikely transmission mechanism, we retained the observation and assumed that infection resulted from exposure in the most recent experiment. Most of the IIPs were directly observed, except for volunteers 52 and 92 who were exposed at least twice. Volunteer 59 was excluded because the IIP could not be completely read (the unreadable observed IIP for several others was confirmed in the text). Five additional observations were reported in the text and not present in the table: four for Filipinos from non-endemic areas and one for an American volunteer (AV 49).

Siler, Hall, and Hitchens, 1926. The experimental human infections by *Ae. aegypti* are reported in Table 2. Those also reported in Table 3 are assumed to have interval-censored IIP observations as they were exposed to multiple potentially infectious feedings.

Schule, 1928. Schule used volunteers for second experiments if they did not become ill within 9-10 days of the first infection experiment, assuming that the IIP was less than 9 days. Because this is precisely what we are trying to estimate, to avoid circular logic, we assume that this is not known. Instead we use interval censoring for those volunteers that were exposed twice.
