# Supplementary material for: The Incubation Periods of Dengue Viruses
Source: PLoS One. 2012 Nov 30;7(11):e50972. doi: 10.1371/journal.pone.0050972 (PMC3511440; doi:10.1371/journal.pone.0050972)
Supplement: Text S2 — Priors (DOCX) [file pone.0050972.s002.docx]

**Text S2:** **Priors**

We found that weakly informative priors improved the sampling process substantially. Most of what we know about the EIP is based on the data used here, so we based the priors on information known before these observations. The earliest report we found speculating explicitly about the EIP, was that of Bancroft ([1](#_ENREF_1)906) who proposed a period of approximately 12 days as had been estimated for yellow fever virus. We set the prior standard deviation for the EIP at half of that, i.e. 6 days. It was also clear early on that once exposed to mosquitoes, humans could become sick with dengue within 4-6 days (Graham 1903). We therefore set the IIP prior to 5 days with a standard deviation of 2.5 days. The mean and variance of each incubation period where then used to calculate the corresponding means for the priors of β_0_, shape, and precision. The standard deviation of each of these priors was set to one third of the respective prior mean, indicating that the value is expected to be greater than zero and not many times greater than the prior mean.

For β_T_, we used a prior mean of zero. We further expect that any effect will be on a scale of less than one tenth of β_0_ per degree of temperature change, so we set the β_T_ prior standard deviation to one third of that value. The mean of the random study effects is zero and its variance should also be limited relative to β_0_. The mean of its prior precision, τ_S_, was therefore set to be equivalent to a standard deviation of one tenth of the β_0_ prior mean; and the τ_S_ prior standard deviation was set to one third of that value. For each serotype dummy variable, the corresponding β prior mean was similarly set to 0 with a prior standard deviation of one tenth of the β_0_ mean.
