# Supplementary material for: The Incubation Periods of Dengue Viruses
Source: PLoS One. 2012 Nov 30;7(11):e50972. doi: 10.1371/journal.pone.0050972 (PMC3511440; doi:10.1371/journal.pone.0050972)
Supplement: Text S3 — Model Comparisons. (DOCX) [file pone.0050972.s003.docx]

**Text S3: Model Comparisons**

**Extrinsic Incubation Period**

To further investigate the relative fit of different models we bootstrapped the observations 100 times, each time fitting them to each model. Because each bootstrap sample is different we ranked the four models on a scale of 1 to 4 (1 being the lowest DIC, 4 being the highest). The rank frequency and average rank are shown in the table below. The log-normal model was most frequently the best model when evaluated by DIC. It was the best fitting model in 93% of the samples, with an average rank of 1.1.

**Extrinsic Incubation period bootstrapped DIC rank**

|  | Rank | | | |  | Average rank |
| --- | --- | --- | --- | --- | --- | --- |
|  | 1 | 2 | 3 | 4 |  |  |
| Exponential | 2 | 8 | 4 | 86 |  | 3.7 |
| Weibull | 5 | 44 | 43 | 8 |  | 2.5 |
| Gamma | 0 | 45 | 49 | 6 |  | 2.6 |
| Log-normal | 93 | 3 | 4 | 0 |  | 1.1 |

**Intrinsic Incubation Period**

**Random Effects.** We first analyzed each dataset – complete, pre-1940, and post-1970 – with all 4 models with and without random effects for study. The results, shown below, show that incorporating random effects improved model fit for the complete and pre-1940 datasets for the Weibull, gamma, and log-normal models.

**DIC with random effects - complete dataset**

|  | Without random effects | With random effects |
| --- | --- | --- |
| Exponential | 830 | 830 |
| Weibull | 534 | 506 |
| Gamma | **506** | **471** |
| Log-normal | **506** | 490 |

**DIC with random effects - pre-1940 dataset**

|  | Without random effects | With random effects |
| --- | --- | --- |
| Exponential | 767 | 768 |
| Weibull | 502 | 476 |
| Gamma | **473** | **445** |
| Log-normal | **473** | 460 |

**DIC with random effects - post-1970 dataset**

|  | Without random effects | With random effects |
| --- | --- | --- |
| Exponential | 60 | 61 |
| Weibull | 33 | 34 |
| Gamma | **30** | **31** |
| Log-normal | 32 | 32 |

**Serotypes.** We then assessed the significance of serotype data for all four models using the complete dataset. As seen in the tables below, the serotype coefficients were all non-significant and their inclusion did not improve model fit as measured by DIC.

**Serotype covariates**

|  | β_DENV-1_ | | β_DENV-2_ | | | β_DENV-3_ | | β_DENV-4_ | | |
| --- | --- | --- | --- | --- | --- | --- | --- | --- | --- | --- |
|  | Mean | 95% CI | | Mean | 95% CI | Mean | 95% CI | | Mean | 95% CI |
| Exponential | 0.0 | -0.3, 0.2 | | 0.0 | -0.3, 0.3 | 0.0 | -0.4, 0.3 | | 0.0 | -0.3, 0.2 |
| Weibull | -0.2 | -0.7, 0.3 | | -0.1 | -0.8, 0.6 | 0.0 | -0.7, 0.7 | | -0.1 | -0.7, 0.4 |
| Gamma | 0.1 | -0.1, 0.3 | | 0.1 | -0.2, 0.4 | -0.1 | -0.3, 0.2 | | 0.0 | -0.2, 0.2 |
| Log-normal | 0.01 | -0.05, 0.07 | | 0.01 | -0.07, 0.09 | -0.01 | -0.09, 0.07 | | 0.01 | -0.06, 0.07 |

**DIC with serotype covariate – complete dataset**

|  | Without serotype data | With serotype data |
| --- | --- | --- |
| Exponential | 830 | 831 |
| Weibull | 506 | 505 |
| Gamma | **471** | 503 |
| Log-normal | 490 | **490** |

**Best fitting model.** The gamma and log-normal models had the lowest DIC in all cases (lowest DIC in bold in the above tables), with the gamma model having the lowest DIC in models including random effects applied to the complete dataset and the pre-1940 dataset. Bootstrap sampling was performed (as described above for the EIP) to substantiate differences in model fit (below). We only analyzed the complete and the pre-1940 datasets as the post-1970 data contained little additional information (as described in the manuscript). The log-normal model had a lower DIC than the gamma model in 89 and 71 out of 100 bootstrap samples for the complete and pre-1940 datasets, respectively.

**Bootstrapped DIC rank – complete dataset**

|  | Rank | | | |  | Average rank |
| --- | --- | --- | --- | --- | --- | --- |
|  | 1 | 2 | 3 | 4 |  |  |
| Exponential | 0 | 0 | 0 | 100 |  | 4 |
| Weibull | 0 | 0 | 100 | 0 |  | 3 |
| Gamma | 11 | 89 | 0 | 0 |  | 1.9 |
| Log-normal | 89 | 11 | 0 | 0 |  | 1.1 |

**Bootstrapped DIC rank - pre-1940 dataset**

|  | Rank | | | |  | Average rank |
| --- | --- | --- | --- | --- | --- | --- |
|  | 1 | 2 | 3 | 4 |  |  |
| Exponential | 0 | 0 | 0 | 100 |  | 4 |
| Weibull | 0 | 0 | 100 | 0 |  | 3 |
| Gamma | 29 | 71 | 0 | 0 |  | 1.7 |
| Log-normal | 71 | 29 | 0 | 0 |  | 1.3 |
